# The Effect of Perceptions of Hookah Harmfulness and Addictiveness on the Age of Initiation of Hookah Use among Population Assessment of Tobacco and Health (PATH) Youth

**DOI:** 10.3390/ijerph19095034

**Published:** 2022-04-21

**Authors:** Arnold E. Kuk, Meagan A. Bluestein, Baojiang Chen, Melissa Harrell, Charles E. Spells, Folefac Atem, Adriana Pérez

**Affiliations:** 1 Michael & Susan Dell Center for Healthy Living, The University of Texas Health Science Center at Houston (UTHealth), Austin, TX 78701, USA; arnold.e.kuk@uth.tmc.edu (A.E.K.); meagan.a.bluestein@uth.tmc.edu (M.A.B.); baojiang.chen@uth.tmc.edu (B.C.); melissa.b.harrell@uth.tmc.edu (M.H.); charles.e.spells@uth.tmc.edu (C.E.S.); 2 Department of Biostatistics and Data Science, The University of Texas Health Science Center at Houston (UTHealth), Austin, TX 78701, USA; 3 Department of Epidemiology, Human Genetics and Environmental Sciences, The University of Texas Health Science Center at Houston (UTHealth), Austin, TX 78701, USA; 4 Department of Biostatistics and Data Science, The University of Texas Health Science Center at Houston (UTHealth), Dallas, TX 75207, USA; folefac.d.atem@uth.tmc.edu

**Keywords:** interval censoring, Cox regression, survival analysis, time to event, tobacco risk perception, waterpipe, narghile, shisha

## Abstract

Despite the negative health consequence of hookah, hookah risk perceptions are misguided among youth. Secondary data analysis of 12–17-year-old never hookah users at their first wave of PATH participation (2013–2019) was performed. The effect of perceptions of hookah harmfulness and addictiveness on the age of initiation ever, past 30-day, and fairly regular hookah use were estimated using interval-censored Cox proportional hazards models. The distribution of the age of initiation of hookah outcomes by perception levels of harmfulness and addictiveness are reported as cumulative incidence and 95% CI. Youth who perceived hookah to be neither harmful nor addictive were 173% more likely to initiate ever, 166% more likely to first report past 30-day use, and 142% more likely to first report fairly regular hookah use at earlier ages compared to youth who considered hookah to be both harmful and addictive. By age 18, 25.5% of youth who perceived hookah as neither harmful nor addictive were estimated to initiate ever hookah use while 9.3% of youth who perceived hookah as harmful and addictive were estimated to initiate ever hookah use. These findings indicate the need to provide prevention and education campaigns to change perceptions of the harmfulness and addictiveness of hookah to delay the age of initiation of hookah use.

## 1. Introduction

Tobacco use is the leading cause of preventable death, accounting for about one in five deaths annually in the U.S.A. [1]. The use of tobacco products (TPs) is generally established during adolescence [1,2], as 9 out of 10 adults who use cigarettes daily initiated its use in adolescence [2]. In 2020, the prevalence of ever hookah use among youth was reported annually for the years 2013–2014 (7.4%), 2014–2015 (6.7%), and 2015–2016 (5.2%) in the US [3]. The Population Assessment of Tobacco and Health (PATH) Study from 2013 to 2017 reported the age of initiation of hookah use outcomes as cumulative incidence. For example, among youth never hookah users in the U.S.A., by age 17, 8.3%, 3.3%, and 1.2% first reported ever, past 30-day, and fairly regular hookah use, respectively [4]. A recent 2020 National Youth Tobacco Survey (NYTS) study indicated that 2.1% of middle and high school students have used hookah in the past 30 days [5], which is troubling considering that hookah often serves as an introductory nicotine product among youth [6]. According to the 2019 NYTS study, 7.1% of youth, representing more than 1.9 million middle and high school students in the U.S., had ever tried hookah [7].

A common misperception is that hookah use is less harmful and less addictive than other forms of tobacco [8,9]. However, studies show that a single hookah session results in consuming amounts of nicotine, tar, and carbon monoxide that are equivalent to smoking 100 cigarettes [10,11,12]. Previous studies also indicate that hookah use increases the risk of oral cancer, heart disease, esophageal cancer, chronic obstructive pulmonary disease, and thrombogenesis [13,14,15,16]. A 2017 study showed that 32% of youth and young adults in their sample perceived hookah to be a lot or a little less harmful than cigarettes [17]. Hookah tobacco contains nicotine and is addictive [8,18], so hookah users’ beliefs about their ability to quit seem misguided [19].

A 2013–2015 cross-sectional study in Washington, D.C., found that youth who viewed hookah as somewhat (AOR: 5.70; 95% CI: 1.37–23.77) or very socially acceptable (AOR: 12.36; 95% CI: 2.61–58.50) had higher odds to report ever hookah use compared to youth who viewed hookah as not socially acceptable [9]. PATH studies from 2013 to 2014 showed that youth who perceived hookah as having “no or little harm” were 2.7 times more likely to initiate ever hookah use one year later compared to youth who perceived hookah as having “a lot of harm” [20], and that youth who were in the “low” category of perceived harmfulness of hookah and youth who were in the “low” and “medium” categories of perceived addictiveness of hookah had higher probabilities of initiating hookah use a year later compared to youth in the “high” categories [21]. While the relationship between hookah use and harm and addiction perceptions is clear, it is unknown how the perceived harmfulness and addictiveness of hookah impact the age of initiation of hookah use outcomes among youth. An earlier age of initiation of hookah can harm the developing brain, affecting learning, memory, and attention [7,22]. In this study, we conducted secondary data analysis of U.S.A. youth never hookah users (12–17 years old at their first wave of participation in PATH), prospectively estimating the impact of perceptions of hookah harmfulness and addictiveness on the age of initiation or first report of three outcomes: (i) ever, (ii) past 30-day, and (iii) fairly regular hookah use. Furthermore, to the best of our knowledge, no previous studies have prospectively examined the interaction of perceptions of harmfulness and addictiveness of hookah use on the age of initiation of hookah use outcomes among U.S.A. youth. For this reason, we analyzed the effect of the interaction between these two perceptions on the age of hookah initiation outcomes and estimated the distribution of the age of initiation of hookah use outcome by each category of the interaction. 

## 2. Materials and Methods

### 2.1. Study Design and Participants

PATH is an ongoing nationally representative longitudinal cohort study of youth and adult tobacco use, collected annually or biannually. Analyses were performed on five waves of PATH-restricted data: wave 1 (12 September 2013–14 December 2014), wave 2 (23 October 2014–30 October 2015), wave 3 (19 October 2015–23 October 2016), wave 4 (1 December 2016–3 January 2018), and wave 5 (1 December 2018–30 November 2019). Family members of PATH participants who were 9–11 years old at wave 1 entered the study at subsequent waves when they turned 12 years old, with 2091, 2045, and 1694 new participants entering the PATH youth study at waves 2–4, respectively. When youth aged up (i.e., turned 18), they were invited to participate in the adult study, with 1915, 1907, and 1900 “aged-up” youth participating in the adult survey in waves 2–4, respectively. At wave 4, a new cohort of participants entered the PATH youth and adult studies, including 3739 “replenished sample” of youth [23]. The original investigators of the PATH study obtained informed parental consent and oral assent from youth participants. IRB approval for this study was obtained from the Committee for the Protection of Human Subjects at the University of Texas Health Science Center at Houston with number HSC-SPH-17-0368.

Two subpopulations were studied. First, youth never hookah users (aged 12–17) at their first wave of PATH participation in waves 1–4 were included in the analysis, resulting in a sample size of *n* = 22,026 participants (N = 40,456,661), including youth who had never heard of hookah. Fifty-eight participants were removed because of their inconsistent age at the follow-up years, reducing the sample size to 21,968 (N = 40,343,800) participants. Using this sample, we examined the effect of the perceptions of hookah harmfulness and addictiveness on the age of initiation of each hookah use outcome. Second, for exploring the interaction effect of perceptions of hookah harmfulness and addictiveness, we removed 7476 participants who had not heard of hookah or who did not answer the perceptions of harmfulness or addictiveness questions or who answered “Do not know”, resulting in a sample size of *n* = 14,492 participants (N = 26,515,180). Perceptions of hookah harmfulness and addictiveness were measured in the wave participants who first entered the PATH study in waves 1–4, and the age of initiation of ever, past 30-day, and fairly regular hookah use outcomes were followed up in waves 2–5.

### 2.2. Measures

#### 2.2.1. Ever, Past 30-Day, and Fairly Regular Hookah Use

Participants at their first wave of PATH participation were asked: “Have you seen or heard of a hookah before this study?”. Participants who reported “yes” were then asked the following question: “Have you smoked tobacco in a hookah, even one or two puffs?”. Participants who reported “yes” were categorized as ever users. Participants who reported “no” to either one of the questions were considered never users. Participants were also asked “When was the last time you smoked tobacco in a hookah, even one or two puffs?”. Participants who responded “Earlier today”, “Not today but sometime in the past 7 days”, and “Not in the past 7 days but sometime in the past 30 days” were categorized as past 30-day users. Finally, participants were asked “Have you ever smoked hookah fairly regularly?”. Participants who responded “yes” were categorized as fairly regular users. For all questions, participants who answered “don’t know” or “refused” were categorized as missing.

#### 2.2.2. Exposures: Perceptions of Hookah Harmfulness and Addictiveness

For each participant, the perception of harmfulness and addictiveness was measured at their first wave of PATH participation (waves 1–4). Because the adult survey did not ask participants the question about hookah addictiveness, we were not be able to create a time-varying measure of the perception of harmfulness and addictiveness [23]. Participants who had seen or heard of hookah were asked: “How much do you think people harm themselves when they smoke hookah?”, with response options including “No harm”, “A little harm”, “Some harm”, “A lot of harm”, “Don’t know”, and “Refused”. Participants who responded “No harm” or “A little harm” were recoded as “No/little harm”. Participants who did not receive this question because they had never heard of hookah were coded as “Never heard of hookah”, resulting in the following categories: “No/little harm”, “Some harm”, “A lot of harm”, “Don’t know”, and “Never heard of hookah”. Participants were also asked “How likely is someone to become addicted to shisha or hookah tobacco?”, with response options including “Very unlikely”, “Somewhat unlikely”, “Neither likely nor unlikely”, “Somewhat likely”, “Very likely”, “Don’t know”, and “Refused”. This variable was recoded as follows: “Very/somewhat unlikely”, “Neither likely nor unlikely”, “Somewhat/very likely”, “Don’t know”, and “Never heard of hookah”. Participants who refused to answer these questions were considered missing.

The interaction between perceptions of harmfulness and addictiveness was assessed based on the dichotomized version of these perception variables. Perception of harmfulness was dichotomized into “A lot of harm” versus “No/little/some harm”. Perception of addictiveness was dichotomized into “Very/somewhat unlikely/neither likely nor unlikely” versus “Somewhat/very likely”. These dichotomized perception variables were then used to create an interaction variable with the following categories: “Neither harmful nor addictive”, “Harmful but not addictive”, “Addictive but not harmful”, and “Both harmful and addictive”.

#### 2.2.3. Covariates

Sex was self-reported as either male or female. Participants’ race was self-reported as White alone, Black alone, Asian alone, and other (including multiracial). Participants’ ethnicity was self-reported as either Hispanic or non-Hispanic. For the few participants who refused to answer these questions, PATH imputed their values at wave 1 using the household information [23]. Following prior studies [1,4,24,25,26,27,28,29,30,31], we combined race and ethnicity into four categories: Non-Hispanic White, Hispanic, Non-Hispanic Black, and Non-Hispanic Other (Asian, multi-race, and other races).

Ever use of tobacco products other than hookah, including cigarettes, e-cigarettes, smokeless tobacco, and cigar products (cigarillos, filtered cigars, and traditional cigars), were measured. For each hookah use outcome, previous ever use of other TPs was identified at the wave prior to initiation of each hookah use outcome. For never hookah users, previous ever use of other tobacco products was identified at the last wave of participation to provide the most up-to-date information.

### 2.3. Age of Initiation of Hookah Use Outcomes

While PATH participants reported hookah use (i.e., ever use, past 30-day use, or fairly regular use) in each wave, asking about the exact date of initiation of each outcome was not feasible. Additionally, participant birthdays were not provided in the restricted-use dataset. This could be problem because a participant who initiated hookah use a few days after participating in wave 1 and a different participant who initiated hookah use a few days before wave 2 would both be shown as initiating ever hookah use in wave 2, even though there is almost a year gap between the two dates of initiation. This implies that using age at each wave to estimate the age of initiation can be imprecise. Instead, we used two variables to estimate the age of initiation of hookah use outcomes: participants’ age at the first wave of PATH participation and the number of weeks between waves based on the first report of each hookah use outcome (i.e., ever use, past 30-day use, or fairly regular use) or the last report of never use. For all participants, the lower age bound was the participants’ age at the first wave of PATH participation plus the number of weeks between waves until the last wave the participant reported non-use of each hookah outcome. For those who initiate or first report hookah use, the upper age bound was the age at the lower bound plus the number of weeks between waves until the first wave the participant reported initiation of each hookah outcome. If a participant remained a never user/non-user of each hookah outcome, then their upper age bound was censored.

### 2.4. Statistical Analysis

To account for the complex survey design of PATH, all analyses used sampling weights assigned at each participants’ first wave of participation (waves 1–4), 100 balanced repeated replicate weights, and Fay’s correction factor, 0.3 [32]. Analyses were conducted on two different subsamples: first, youth never hookah users, including those who had never heard of hookah, to examine the effect of their perception of hookah harmfulness and addictiveness, separately, on the age of initiation, and second, youth never hookah users who have heard of hookah and who responded to questions measuring their perceptions of hookah harmfulness and addictiveness to examine the effect of the interaction of the two perceptions on the age of initiation of hookah use outcomes. For each sample, weighted summary statistics for all variables are provided. Weighted interval-censored multivariable Cox proportional hazard models [33,34] with a piecewise constant baseline hazard function were used to examine differences in the age of initiation of each hookah use outcome by each exposure variable, while controlling for sex, race/ethnicity, previous ever use of cigarettes, previous ever use of e-cigarettes, previous ever use of cigar products, and previous ever use of smokeless tobacco. Adjusted hazard ratios (AHR) and corresponding 95% confidence intervals (CI) are reported. In the first subpopulation, 6 adjusted models were fit (3 outcomes × 2 exposures: Models 1–6), and in the second subpopulation, 3 adjusted models were fit (3 outcomes × 1 interaction exposure: Models 7–9). In the second subpopulation, because the interaction between perceptions of harmfulness and addictiveness regarding the age of initiation of hookah use outcomes was significant, weighted interval-censored survival analyses [35,36] were used to estimate the age of initiation of each hookah use outcome for each exposure interaction category. The hazard function and 95% CI for each outcome were estimated using the Turnbull non-parametric estimator, and are reported as cumulative incidence in percentages, which are presented in the figure. Assessment of the heterogeneity of effects was conducted to compare the observed interaction effect and expected effect using the dichotomized versions of the risk perceptions variables (harmfulnessAHR × addictivenessAHR) [37]. All statistical analyses were conducted using SAS version 9.4. A type I error level of 0.05 was used to declare significant results.

## 3. Results

Table 1 shows the demographic characteristics of the first subpopulation. Their average age at the first wave of participation was 13.7, 56.7% entered the study in 2013–2014, 51.3% were males, 22.8% were Hispanic, and 17.4% and 28.9% had used cigarettes and e-cigarettes, respectively, prior to hookah initiation.

Table 2 shows the results for multivariable Cox models for the associations between (i) perceptions of hookah harmfulness and (ii) perceptions of hookah addictiveness regarding the age of initiation of each hookah use outcome. After controlling for sex, race/ethnicity, and previous ever use of the other four tobacco products, the results show that youth who perceived hookah as having “no/little harm”, “some harm”, or responded “don’t know” were 166% (AHR: 2.66; 95% CI: 2.29–3.09), 59% (AHR: 1.59; 95% CI: 1.41–1.80), or 80% (AHR: 1.80; 95% CI: 1.29–2.52) more likely to initiate ever hookah use at earlier ages compared to youth who perceived hookah as having “a lot of harm”. As expected, youth who had never heard of hookah had a decreased risk of initiating ever hookah use at earlier ages compared to youth who perceived hookah as having “a lot of harm” (see Model 1). Similar results were observed for the age of initiation of first report of past 30-day hookah use except for those who responded “don’t know”, which was not significant (see Model 2). For fairly regular hookah use, only youth who perceived hookah as having “no/little harm” had an increased risk of initiating fairly regular hookah use at earlier ages compared to youth who perceived hookah as having “a lot of harm” (see Model 3).

Compared to youth who perceived the addictiveness of hookah to be “somewhat/very likely”, youth who perceived the addictiveness of hookah to be “very/somewhat unlikely”, “neither likely nor unlikely”, or responded “don’t know” were 98% (AHR: 1.98; 95% CI: 1.67–2.33), 62% (AHR: 1.62; 95% CI: 1.45–1.81), or 39% (AHR: 1.39; 95% CI: 1.15–1.69) more likely to initiate ever hookah use at earlier ages, and youth who had never heard of hookah had a lower risk of initiating ever hookah use at earlier ages (see Model 4). Similarly, youth who perceived the addictiveness of hookah to be “very/somewhat unlikely” or “neither likely nor unlikely” had a higher risk of first reporting past 30-day and fairly regular hookah use at earlier ages compared to youth who perceived the addictiveness of hookah to be “somewhat/very likely” (see Models 5 and 6). As expected, youth who had never heard of hookah had a lower risk of first reporting past 30-day hookah use at earlier ages. 

Table 3 shows the demographic characteristics of the second subpopulation of youth who were never users of hookah at their first wave of PATH participation. Their average age at the first wave of participation was 13.9, 46.9% entered the study between 2013 and 2014, 50.1% were males, 51.2% were non-Hispanic White, 24.6% were Hispanic, and 44.7% perceived hookah use as both harmful and addictive. Previous other tobacco use varied from 5.5% for smokeless tobacco to 29.9% for e-cigarettes.

Table 4 reports the adjusted hazard ratios from the multivariable Cox analyses for the interaction effect of perceptions of harmfulness and addictiveness on the age of initiation of each hookah use outcome, after controlling for sex, race/ethnicity, and previous ever use of four other tobacco products. Youth who perceived hookah as “harmful but not addictive”, “addictive but not harmful”, and “neither harmful nor addictive” were 55% (AHR: 1.55; 95% CI: 1.18–2.03), 81% (AHR: 1.81; 95% CI: 1.56–2.10), and 173% (AHR: 2.73; 95% CI: 2.35–3.16) more likely to initiate ever hookah use at earlier ages compared to youth who perceived hookah to be “both harmful and addictive” (see Model 7). Youth who perceived hookah as “neither harmful nor addictive” and “addictive but not harmful” were 66% (AHR: 1.66; 95% CI: 1.32–2.09) and 166% (AHR: 2.66; 95% CI: 2.10–3.37) more likely to first report past 30-day hookah use at earlier ages compared to youth who perceived hookah as “both harmful and addictive” (see Model 8). Finally, youth who perceived hookah as “neither harmful nor addictive” were 142% (AHR: 2.42; 95% CI: 1.46–4.30) more likely to first report fairly regular hookah use at earlier ages compared to youth who perceived hookah as “both harmful and addictive (see Model 9).

When we examined the heterogeneity of effects in the interactions between perceptions of harmfulness and addictiveness on the age of hookah initiation, we compared the sum of the perceptions’ independent effect with their observed joint effect (harmfulnessAHR × addictivenessAHR). Our analysis found that, for all ages of initiation or first reports of hookah use outcomes, the observed joint effect of perceptions of harmfulness and addictiveness was smaller than the expected effect, indicating an antagonistic rather than synergistic interaction. 

**Figure 1:**
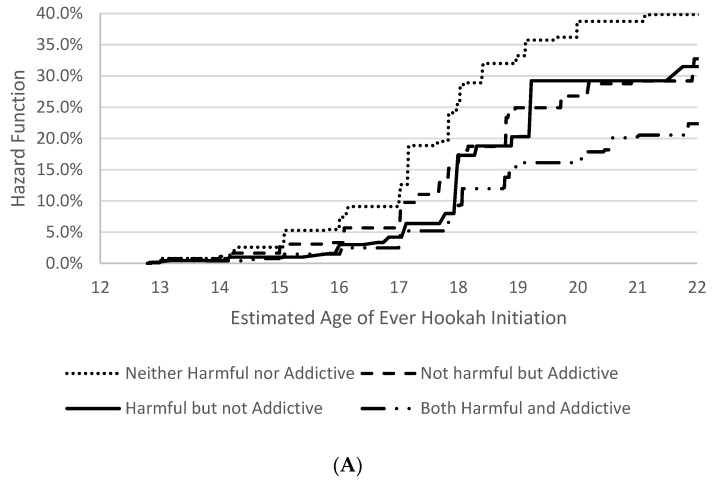

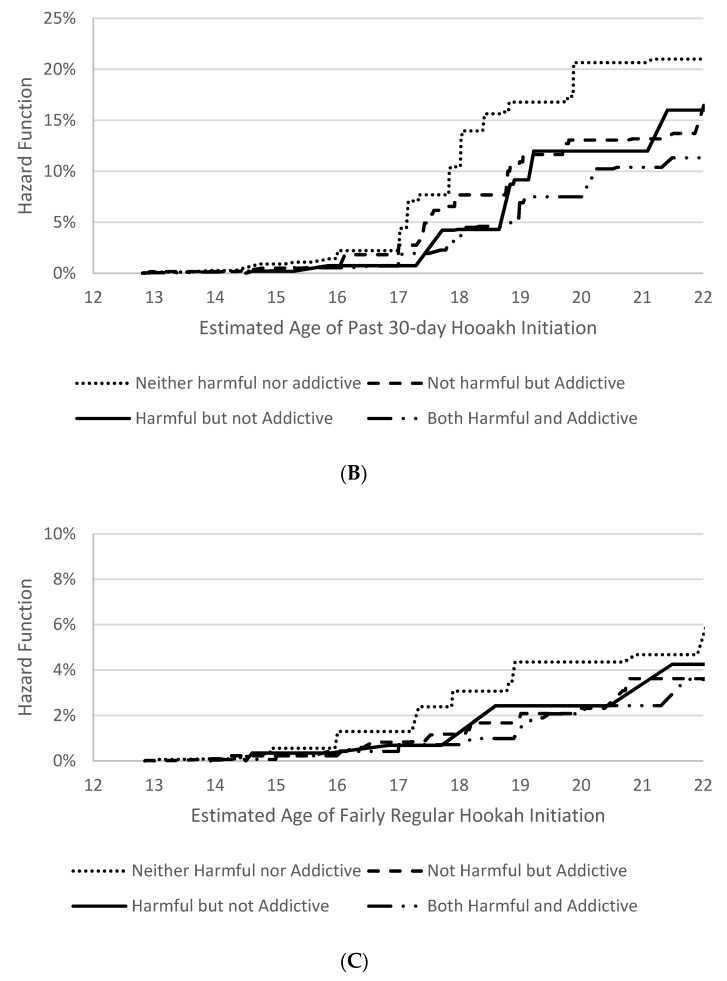
Hazard functions (cumulative incidence in percentage) of the age of initiation of (**A**) ever use, (**B**) past 30-day use, and (**C**) fairly regular use of hookah by perception levels.

Because the interaction of perceptions of harmfulness and addictiveness of hookah was associated with the age of initiation of hookah use outcomes, the distribution of the age of initiation of each hookah outcome by each level of the interaction is presented in Figure 1, which represents the estimated hazard function of each of the ages of initiation of hookah use outcomes by each interaction level. By age 18, 9.3% of youth who perceived hookah as “both harmful and addictive” were estimated to initiate ever hookah use, whereas 17.3% of youth who perceived hookah as “harmful but not addictive”, 18.1% of youth who perceived hookah as “addictive but not harmful”, and 25.5% of youth who perceived hookah as “neither harmful nor addictive” were estimated to initiate ever hookah use. By age 18, while only 3.7% of youth who perceived hookah as “both harmful and addictive” were estimated to first report past 30-day hookah use, 7.7% of youth who perceived hookah as “addictive but not harmful” and 10.4% of youth who perceived hookah as “neither harmful nor addictive” were estimated to first report past 30-day hookah use. Finally, 3.1% of youth who perceived hookah as “neither harmful nor addictive” were estimated to initiate fairly regular hookah use by age 18, while only 1.0% of youth who perceived hookah as “both harmful and addictive” were estimated to initiate fairly regular hookah use by age 18. The highest increase in ever hookah use among youth who perceived hookah as “neither harmful nor addictive” or youth who perceived hookah as “addictive but not harmful” or youth who perceived “harmful but not addictive” was between 17 and 18 years old, indicating the small window of opportunity to educate youth about the harmfulness and addictiveness of hookah before they initiate hookah.

## 4. Discussion

This paper examines how perceptions of harmfulness and addictiveness of hookah use among youth have differential effects on their age of initiation or first reporting of ever, past 30-day, and fairly regular hookah use after controlling for sex, race/ethnicity, previous ever use of cigarettes, previous ever use of e-cigarettes, previous ever use of cigar products and previous ever use of smokeless tobacco. In a previous publication, we reported the association of the individual effect of sex and race/ethnicity with the age of initiation of hookah use outcomes [4]. Our results are consistent with prior studies examining the associations between perceptions of harmfulness and addictiveness of hookah and hookah initiation. Prior studies of hookah use have warned that the low perceptions of harmfulness and addictiveness among youth may lead to subsequent hookah initiation [16,17,27]. Our results extend these findings by examining these risk perceptions regarding the ages of hookah initiation outcomes. PATH data from 2013 to 2015 show that youth who responded “don’t know” to hookah risk perception questions had slightly higher probabilities of trying hookah a year later [21]. Similarly, we show that youth who responded “don’t know” to hookah perception questions were more likely to initiate ever hookah use at earlier ages compared to youth who perceived hookah as having “a lot of harm” or youth who perceived the addictiveness of hookah to be “somewhat/very likely”, suggesting the importance of educating youth about the harmfulness and addictiveness of hookah use [13]. As expected, youth who had never heard of hookah were less likely to initiate ever or report past 30-day hookah use at earlier ages compared to youth who perceived hookah as having “a lot of harm” or youth who perceived the addictiveness of hookah to be “somewhat/very likely”. Future research should examine when and how youth become aware of hookah products to help identify at what ages education campaigns can be helpful.

This study further examined the interaction effect of perceived hookah harmfulness and addictiveness and found an antagonistic effect between the perceptions of harmfulness and addictiveness, suggesting that the effect of perceptions of harmfulness and addictiveness on hookah initiation is not additive. This implies that it may be more effective for education and prevention communication campaigns to focus exclusively on the potential harmfulness or potential addictiveness associated with hookah use, separately. Given the large hazard ratios reported here for perception of harmfulness, it may be most prudent to focus on increasing young people’s knowledge about the negative health effects of hookah use in these campaigns. 

Finally, this study also reported the estimated age of hookah initiation by each interaction category of these risk perceptions. Our results indicate that the highest increase in hookah initiation for youth who perceived hookah to be “neither harmful nor addictive” occurs between 17 and 18 years old. Identifying this short window of opportunity is extremely important given that education campaigns should happen between 17 and 18 years old to decrease past 30-day hookah use by 8% among youth who perceived hookah as “neither harmful nor addictive”. This information can help inform education and prevention communication campaigns with specific age targets for the education campaigns to prevent early age of initiation of hookah use outcomes.

### Strength and Limitations

This study is the first to examine the association between perceptions of harmfulness and addictiveness of hookah use and the age of initiation of hookah outcomes among youth. We used the first five waves of PATH (2013–2019) of a nationally representative study to perform prospective analyses estimating the age of initiation of hookah use outcomes, which no prior studies have done before. In addition, to the best of our knowledge, we are the first to examine the effect of the interaction between perceptions of hookah harmfulness and addictiveness on the age of initiation of hookah use. This study is not without limitations. While some researchers may see it as a limitation that we combined the categories for “no/little/some harm” for the interaction variable, we consider that “a lot of harm” is a clear indication of beliefs about the harmfulness of hookah. Similarly, we considered “Somewhat/very likely” to be a clear indication of perception of hookah addictiveness. Another limitation is that the estimation of the age of initiation of fairly regular hookah use can be seen as a subjective measure as each participant has their own interpretation of what constitutes “fairly regular hookah use”. We overcame this limitation by estimating the age of initiation of ever hookah use and the age of first report of past 30-day hookah use as they are standard measures for tobacco use. Finally, although we presented the hazard ratios of sex, race/ethnicity, and prior use of other tobacco products, they are not discussed as they were included as control variables for the effect of perception of harmfulness and addictiveness of hookah.

## 5. Conclusions

This study provides evidence that youth with misguided perceptions of hookah harmfulness and addictiveness are at a higher risk of initiating hookah use at earlier ages compared to youth with more accurate perceptions of hookah harmfulness and addictiveness. These findings suggest the need to provide prevention and education campaigns to guide youth and inform them about the harmfulness and addictiveness of hookah use to delay the age of initiation. Furthermore, we provide the age “windows” before hookah initiation needed for these campaigns.

## Figures and Tables

**Table 1:** Demographic characteristics of Population Assessment of Tobacco and Health (PATH) youth (aged 12–17) never hookah users at their first wave of participation.

Total		*n* = 21,968; N = 40,343,800	
		*n* (N)	Weighted % (SE)
First wave of PATH ^¥^ participation	Wave 1 (2013–2014)	12,585 (22,894,057)	56.7% (0.11)
	Wave 2 (2014–2015)	2071 (4,079,970)	10.1% (0.08)
	Wave 3 (2015–2016)	2012 (4,142,408)	10.3% (0.11)
	Wave 4 (2016–2017)	5300 (9,227,366)	22.9% (0.10)
Age at entry into study (SE)	Weighted mean (SE)	13.7 (0.005)	
Sex	Female	10,587 (19,612,356)	48.6% (0.12)
	Male	11,372 (20,711,496)	51.3% (0.12)
	Missing	9 (19,949)	0.1% (0.02)
Race/ethnicity	Non-Hispanic White	10,492 (21,491,690)	53.3% (0.14)
	Hispanic	6332 (9,250,260)	22.9% (0.11)
	Non-Hispanic Black	3000 (5,520,016)	13.7% (0.08)
	Non-Hispanic Other ^1^	2098 (3,999,530)	9.9% (0.10)
	Missing	46 (82,304)	0.2% (0.04)
Ever use of cigarettes prior to	Yes	3922 (7,036,954)	17.4% (0.37)
hookah initiation	No	18,026 (33,271,260)	82.5% (0.37)
	Missing	20 (35,586)	0.1% (0.02)
Ever use of e-cigarettes prior to	Yes	6317 (11,670,433)	28.9% (0.47)
hookah initiation	No	15,463 (28,276,108)	70.1% (0.47)
	Missing	188 (397,259)	1.0% (0.07)
Ever use of any cigar products prior	Yes	2557 (4,655,341)	11.5% (0.27)
to hookah initiation	No	19,206 (35,302,536)	87.5% (0.28)
	Missing	205 (24,938)	1.0% (0.06)
Ever use of smokeless tobacco prior	Yes	1224 (2,292,428)	5.7% (0.23)
to hookah initiation	No	20,595 (37,777,447)	93.6% (0.25)
	Missing	149 (273,925)	0.7% (0.06)
Perception of harmfulness	No/little harm	2293 (4,155,555)	10.3% (0.30)
	Some harm	4721 (8,634,302)	21.4% (0.37)
	A lot of harm	8004 (14,723,395)	36.5% (0.36)
	Don’t know	384 (738,425)	1.8% (0.10)
	Not heard of hookah	6554 (12,072,899)	29.9% (0.48)
	Missing	12 (19,224)	
Perception of addictiveness	Very/somewhat unlikely	1358 (2,461,298)	6.1% (0.17)
	Neither likely nor unlikely	2043 (3,707,919)	9.2% (0.24)
	Somewhat/very likely	11,228 (20,605,841)	51.1% (0.39)
	Don’t know	769 (1,464,765)	3.6% (0.15)
	Not heard of hookah	6554 (12,072,899)	29.9% (0.48)
	Missing	16 (31,077)	

^¥^ Population Assessment of Tobacco and Health (PATH). Study data reprinted with permission from the United States Department of Health and Human Services (National Addiction & HIV Data Archive Program, 2021). Restricted file received disclosure to publish: 11 January 2022. ^1^ Non-Hispanic Other includes Asian, multi-race, etc.

**Table 2:** Multivariable hazard ratios (95% confidence intervals) for the association between perceptions of harmfulness and addictiveness with the age of initiation of hookah use outcomes.

	Ever Use ^¥^	Past 30-Day Use ^¥^	Fairly Regular Use ^¥^
	Model 1	Model 2	Model 3
Perception of Harmfulness			
A lot of harm	1.00	1.00	1.00
Some harm	**1.59 (1.41–1.80)**	**1.41 (1.13–1.76)**	1.29 (0.84–1.97)
No/little harm	**2.66 (2.29–3.09)**	**2.74 (2.23–3.38)**	**2.45 (1.56–3.86)**
Don’t know	**1.80 (1.29–2.52)**	1.53 (0.92–2.54)	1.88 (0.52–6.76)
Never heard of hookah	**0.69 (0.58–0.81)**	**0.67 (0.52–0.85**)	0.83 (0.52–1.33)
Sex			
Female	1.00	1.00	1.00
Male	**0.91 (0.83–0.99)**	0.93 (0.80–1.07)	0.81 (0.59–1.11)
Race/ethnicity			
Non-Hispanic White	1.00	1.00	1.00
Hispanic	1.14 (0.98–1.33)	**1.38 (1.14–1.67)**	**1.75 (1.26–2.43)**
Non-Hispanic Black	**1.16 (1.02–1.32)**	**1.58 (1.29–1.94)**	1.48 (0.95–2.30)
Non-Hispanic Other ^1^	1.09 (0.91–1.29)	**1.52 (1.19–1.93)**	1.16 (0.65–2.07)
Ever use of other tobacco products prior to hookah initiation			
Cigarette use—No	1.00	1.00	1.00
Cigarette use—Yes	**1.37 (1.19–1.58)**	1.22 (0.98–1.53)	**1.83 (1.20–2.79)**
E-cigarette use—No	1.00	1.00	1.00
E-cigarette use—Yes	**0.71 (0.64–0.79)**	**0.78 (0.64–0.94)**	0.77 (0.55–1.08)
Any cigar use—No	1.00	1.00	1.00
Any cigar use—Yes	0.92 (0.79–1.06)	0.93 (0.73–1.18)	0.89 (0.57–1.41)
Smokeless tobacco use—No	1.00	1.00	1.00
Smokeless tobacco use—Yes	1.06 (0.88–1.29)	1.15 (0.88–1.50)	1.13 (0.60–1.08)
	**Model 4**	**Model 5**	**Model 6**
Perception of Addictiveness			
Somewhat/very likely	1.00	1.00	1.00
Neither likely nor unlikely	**1.62 (1.45–1.81)**	**1.65 (1.36–2.01)**	**1.71 (1.12–2.62)**
Very/somewhat unlikely	**1.98 (1.67–2.33)**	**1.97 (1.61–2.41)**	**1.93 (1.19–3.13)**
Don’t know	**1.39 (1.15–1.69)**	1.24 (0.89–1.72)	1.02 (0.44–2.34)
Never heard of hookah	**0.53 (0.47–0.61)**	**0.53 (0.44–0.66)**	0.71 (0.47–1.08)
Sex			
Female	1.00	1.00	1.00
Male	**0.89 (0.81–0.97)**	0.89 (0.78–1.04)	0.81 (0.59–1.11)
Race/ethnicity			
Non-Hispanic White	1.00	1.00	1.00
Hispanic	1.15 (0.98–1.34)	**1.39 (1.15–1.69)**	**1.77 (1.27–2.47)**
Non-Hispanic Black	**1.19 (1.04–1.37)**	**1.63 (1.31–2.02)**	1.49 (0.95–2.36)
Non-Hispanic Other ^1^	1.07 (0.89–1.29)	**1.50 (1.17–1.94)**	1.17 (0.66–2.08)
Ever use of other tobacco products prior to hookah initiation			
Cigarette use—No	1.00	1.00	1.00
Cigarette use—Yes	**1.35 (1.18–1.57)**	1.19 (0.95–1.51)	**1.78 (1.15–2.74)**
E-cigarette use—No	1.00	1.00	1.00
E-cigarette use—Yes	**0.72 (0.65–0.81)**	**0.79 (0.65–0.96)**	0.78 (0.56–1.09)
Any cigar use—No	1.00	1.00	1.00
Any cigar use—Yes	0.93 (0.80–1.08)	0.93 (0.73–1.19)	0.89 (0.56–1.40)
Smokeless tobacco use—No	1.00	1.00	1.00
Smokeless tobacco use—Yes	1.07 (0.89–1.29)	1.17 (0.89–1.52)	1.16 (0.62–2.17)

^¥^ Population Assessment of Tobacco and Health (PATH). Study data reprinted with permission from the United States Department of Health and Human Services (National Addiction & HIV Data Archive Program, 2021). Restricted file received disclosure to publish: 11 January 2022. ^1^ Non-Hispanic Other includes Asian, multi-race, etc. Bolded number represents statistical significance < 0.05 type I error level.

**Table 3:** Demographic characteristics of PATH youth (aged 12–17) at their first wave of PATH participation (2013–2018) of the second subpopulation.

Total		*n* = 14,492; N = 26,515,180	
		*n* (N)	Weighted % (SE)
First wave of PATH ^¥^ participation	Wave 1 (2013–2014)	6824 (12,429,639)	46.9% (0.29)
	Wave 2 (2014–2015)	703 (1,389,820)	5.2% (0.17)
	Wave 3 (2015–2016)	1872 (3,838,256)	14.5% (0.19)
	Wave 4 (2016–2017)	5093 (8,857,465)	33.4% (0.27)
Age at entry into study (SE)	Weighted mean (SE)	13.9 (0.009)	
Sex	Female	7163 (13,290,977)	49.8% (0.27)
	Male	7326 (13,216,365)	50.1% (0.27)
	Missing	3 (7838)	0.1% (0.02)
Race/ethnicity	Non-Hispanic White	6606 (13,580,180)	51.2% (0.29)
	Hispanic	4435 (6,515,941)	24.6% (0.27)
	Non-Hispanic Black	1980 (3,644,534)	13.7% (0.21)
	Non-Hispanic Other ^1^	1445 (2,730,255)	10.3% (0.19)
	Missing	26 (44,270)	0.2% (0.04)
Ever use of cigarettes prior to hookah initiation	Yes	2689 (4,802,141)	18.1% (0.42)
	No	11,794 (21,699,339)	81.8% (0.42)
	Missing	9 (13,700)	0.1% (0.02)
Ever use of e-cigarettes prior to hookah initiation	Yes	4303 (7,903,090)	29.8% (0.54)
	No	10,022 (18,258,492)	68.9% (0.53)
	Missing	167 (353,598)	1.3% (0.11)
Ever use of any cigar products prior to hookah initiation	Yes	1783 (3,242,870)	12.2% (0.31)
	No	12,635 (23,134,768)	87.3% (0.32)
	Missing	74 (137,542)	0.5% (0.06)
Ever use of smokeless tobacco prior to hookah initiation	Yes	776 (1,455,018)	5.5% (0.27)
	No	13,619 (24,887,149)	93.9% (0.28)
	Missing	97 (173,014)	0.6% (0.06)
Interaction of perceptions of harmfulness and addictiveness of hookah	Neither harmful nor addictive	2508 (4,550,031)	17.2% (0.39)
	Addictive but not harmful	4258 (7,778,990)	29.3% (0.45)
	Harmful but not addictive	856 (1,552,431)	5.9% (0.20)
	Both harmful and addictive	6870 (12,633,727)	47.6% (0.61)

^¥^ Population Assessment of Tobacco and Health (PATH). Study data reprinted with permission from the United States Department of Health and Human Services (National Addiction & HIV Data Archive Program, 2021). Restricted file received disclosure to publish: 11 January 2022. ^1^ Non-Hispanic Other includes Asian, multi-race, etc.

**Table 4:** Multivariable hazard ratios (95% confidence interval) for the association between the interaction of perceptions of harmfulness and addictiveness with the age of hookah initiation outcomes.

	Ever Use ^¥^	Past 30-Day Use ^¥^	Fairly Regular Use ^¥^
	Model 7	Model 8	Model 9
Interaction of perceptions of hookah harmfulness and addictiveness			
Both harmful and addictive	1.00	1.00	1.00
Harmful but not addictive	**1.55 (1.18–2.03)**	1.28 (0.85–1.94)	1.44 (0.58–3.59)
Addictive but not harmful	**1.81 (1.56–2.10)**	**1.66 (1.32–2.09)**	1.47 (0.89–2.43)
Neither harmful nor addictive	**2.73 (2.35–3.16)**	**2.66 (2.10–3.37)**	**2.42 (1.46–4.03)**
Sex			
Female	1.00	1.00	1.00
Male	**0.90 (0.82–0.99)**	0.95 (0.81–1.11)	0.75 (0.51–1.10)
Race/ethnicity			
Non-Hispanic White	1.00	1.00	1.00
Hispanic	1.10 (0.94–1.29)	**1.37 (1.11–1.68)**	**1.67 (1.14–2.44)**
Non-Hispanic Black	1.11 (0.95–1.29)	**1.52 (1.18–1.95)**	1.08 (0.57–2.03)
Non-Hispanic Other ^1^	1.12 (0.91–1.37)	**1.63 (1.24–2.14)**	1.30 (0.70–2.42)
Previous ever use of other tobacco products			
Cigarette use—No	1.00	1.00	1.00
Cigarette use—Yes	**1.18 (1.02–1.38)**	1.02 (0.79–1.31)	1.44 (0.89–2.34)
E-cigarette use—No	1.00	1.00	1.00
E-cigarette use—Yes	**0.73 (0.65–0.83)**	**0.79 (0.63–0.98)**	**0.68 (0.47–0.99)**
Any cigar use—No	1.00	1.00	1.00
Any cigar use—Yes	0.92 (0.79–1.07)	0.91 (0.69–1.20)	0.92 (0.55–1.53)
Smokeless tobacco use—No	1.00	1.00	1.00
Smokeless tobacco use—Yes	1.03 (0.82–1.29)	1.12 (0.79–1.57)	1.52 (0.78–2.97)

^¥^ Population Assessment of Tobacco and Health (PATH). Study data reprinted with permission from the United States Department of Health and Human Services (National Addiction & HIV Data Archive Program, 2021). Restricted file received disclosure to publish: 11 January 2022. ^1^ Non-Hispanic Other includes Asian, multi-race, etc. Bolded number represents statistical significance < 0.05 type I error level.

## Data Availability

All the data from waves 1–5 are available from the Population Assessment of Tobacco and Health (PATH) Study (United States) Restricted-Use Files. Inter-university Consortium for Political and Social Research (distributor), 21 June 2020. https://doi.org/10.3886/ICPSR36231.v27 accessed on 14 October 2021. Researchers can apply for access to the restricted-use datasets from the Inter-university Consortium for Political and Social Research (ICPSR) at the University of Michigan. To access data in the Virtual Data Enclave (VDE), a Restricted Data Use Agreement (RDUA) must be established between the University of Michigan and the researcher’s institution. Data are provided via ICPSR’s VDE. For further information, please reference the VDE Guide to learn about the application process, about using the VDE, and how to request disclosure review of VDE output located here: https://www.icpsr.umich.edu/web/pages/NAHDAP/vde/index.html accessed on 14 October 2019. Obtaining results using the restricted-use datasets requires a disclosure process with protocols set by ICPSR. When a researcher logs on to the VDE, a virtual machine is launched on the researcher’s own desktop but operates from a server at ICPSR. The virtual machine is isolated from the researcher’s physical desktop computer—users cannot download or upload files or parts of files from or to the VDE; print VDE contents from a printer; or email, copy, or otherwise move files in or out of the VDE computing environment, either accidentally or intentionally. Results are only disclosed by ICPSR after programs have been checked for accuracy and results have been replicated.
